# Early childhood antibiotic exposure, gut microbial balance and immune dysregulation

**DOI:** 10.6026/973206300221144

**Published:** 2026-02-28

**Authors:** Sonali Rode, R Someshwaran, V Harsh Salankar

**Affiliations:** 1 Department of Pharmacology, Government Medical College, Seoni, Madhya Pradesh, India; 2 Department of Microbiology, PSG Institute of Medical Sciences and Research, Coimbatore, Tamil Nadu, India; 3 Department of Pharmacology, N.K.P. Salve Institute of Medical Sciences & Research Centre and Lata Mangeshkar Hospital, Nagpur, Maharashtra, India

**Keywords:** Allergies, antibiotics, gut microbiota, immune dysregulation, microbial diversity

## Abstract

Antibiotic exposure in early childhood has been linked to lasting changes in gut microbial balance and immune system dysregulation, contributing to an increased risk of immune-related diseases. Therefore, it is of interest to investigate the long-term effects of early- life antibiotic use on gut microbiota composition, immune markers and the prevalence of conditions such as asthma and food allergies. Fecal and blood samples were analyzed to assess microbial diversity and immune function in antibiotic-exposed and non-exposed children. Results showed significant differences in microbial diversity, inflammatory cytokine levels and immune markers between the groups. These findings advance knowledge by highlighting the critical role of early antibiotic exposure in shaping immune health and chronic disease susceptibility later in life.

## Background:

Antibiotics are commonly prescribed to treat infections in early childhood, often without fully understanding the long-term consequences of their use. While antibiotics are crucial for combating bacterial infections, their overuse or misuse, especially in early life, can disrupt the delicate balance of the gut microbiota, leading to lasting alterations in microbial composition [1]. The gut microbiota, consisting of trillions of microorganisms, plays a vital role in the development and regulation of the immune system. It is increasingly recognized that the microbiota does not only contribute to digesting food but also plays a fundamental role in the body's immune responses [2]. Therefore, disturbances in the microbiota due to antibiotic exposure could potentially lead to immune system dysregulation, contributing to a wide range of health issues later in life. The early years of life are crucial for the establishment of a stable and diverse gut microbiota, which helps train the immune system to distinguish between harmful pathogens and harmless substances. Disruptions in this early microbial environment, caused by factors such as antibiotic use, can impair the immune system's ability to function properly [3]. Antibiotics, particularly broad- spectrum types, have been shown to reduce microbial diversity in the gut and promote the growth of pathogenic bacteria, potentially leading to long-term immune dysfunction. This dysbiosis, or microbial imbalance, can increase susceptibility to a variety of health problems, such as allergies, asthma, autoimmune diseases and chronic inflammatory conditions [4]. Studies have shown that antibiotic exposure during early childhood can alter the gut microbiota in ways that affect immune system development. For example, antibiotics can lead to a reduction in beneficial microbes such as *Bifidobacteria* and *Lactobacilli,* which are essential for the maintenance of a healthy immune system [5]. In contrast, the abundance of harmful microbes, such as *Clostridia* and *Enterococci*, may increase. These changes can result in an overactive immune response, leading to conditions like allergies and autoimmune disorders. The altered microbial environment may also impair the gut's ability to regulate inflammatory responses, leading to chronic inflammation, which is a key feature of many autoimmune diseases [6]. Moreover, early antibiotic exposure has been linked to an increased risk of diseases that are associated with immune system dysregulation. Research suggests that children who were exposed to antibiotics during infancy are more likely to develop conditions such as asthma, food allergies and inflammatory bowel disease (IBD) later in life [7]. For instance, a study found that children who were given antibiotics within the first six months of life had an increased risk of developing asthma and allergic rhinitis. Similarly, other research has demonstrated that antibiotic use in early childhood may alter the gut-brain axis, potentially contributing to the development of neurodevelopmental disorders such as autism spectrum disorder (ASD) and attention-deficit hyperactivity disorder (ADHD) [8]. The long-term consequences of early antibiotic exposure on gut microbial balance and immune dysregulation have garnered increasing attention in recent years. With the rise in antibiotic-resistant infections and the growing concern about the impact of antibiotics on human health, it is crucial to understand how early exposure to these drugs might shape the microbiota and immune system, influencing long-term health outcomes [9]. Therefore, it is of interest to develop strategies to mitigate the negative effects of antibiotics on microbial diversity, as well as for informing healthcare practices, particularly in the prescribing of antibiotics to young children.

## Methodology:

This research aimed to explore the lasting impact of early childhood antibiotic exposure on gut microbial balance and immune dysregulation. A longitudinal cohort study design was used to assess the gut microbiota composition and immune system function in children who were exposed to antibiotics during early childhood and compare these outcomes to children who were not exposed. The study collected baseline data on gut microbiota composition, immune markers and health outcomes and followed participants over a period of 5 to 10 years to assess the long-term effects of early antibiotic exposure.

## Study design:

The study employed a longitudinal, observational cohort design with two groups: children who had been exposed to antibiotics early in life and children who had not been exposed. The aim was to track changes in gut microbial composition, immune function and the subsequent development of immune-related diseases. The study was conducted in two phases: a baseline phase, followed by a follow-up phase to track health outcomes over time.

## Participants:

Participants were selected from a cohort of children aged 3-5 years who were enrolled in local pediatric clinics or community health centers. The study included two main groups:

[1] Antibiotic-Exposed Group: Children who received at least one course of antibiotics before the age of 2 years.

[2] Non-Antibiotic-Exposed Group: Children who had never been prescribed antibiotics before the age of 2 years.

## Inclusion criteria:

[1] Children aged 3 to 5 years.

[2] Parents/guardians of the child provided informed consent.

[3] Children who had either been exposed to antibiotics or not exposed, based on medical records.

## Exclusion criteria:

[1] Children with chronic medical conditions, such as autoimmune diseases, that could confound the results.

[2] Children with a history of major gastrointestinal surgeries or conditions affecting gut health.

[3] Children with known allergies or immune disorders at the time of recruitment.

## Data collection:

Data were collected at baseline and during follow-up assessments at 6-month intervals for 3 years. The data included:

## Gut microbial composition:

Fecal samples were collected from each participant to assess the composition of gut microbiota. DNA was extracted from stool samples and high-throughput sequencing (16S rRNA sequencing) was performed to identify and quantify microbial species. The diversity and abundance of gut microbiota were assessed, focusing on the relative proportions of beneficial bacteria (e.g., *Bifidobacterium *, *Lactobacillus*) and pathogenic bacteria (e.g., *Clostridium*, *Escherichia coli*).

## Immune system markers:

Blood samples were collected to measure immune system function, including the levels of inflammatory cytokines (e.g., TNF-α, IL-6), immune cell profiles (e.g., T cells, B cells) and markers of immune activation or regulatory responses (e.g., TGF-β, IL-10). Serum immunoglobulin levels (IgA, IgG, IgM) were also measured as markers of immune system status.

## Health outcomes:

Parents completed surveys on their child's health status, including any history of allergies, asthma, food allergies, eczema, or autoimmune diseases. A validated questionnaire, such as the Pediatric Asthma Quality of Life Questionnaire (PAQLQ), was used to assess the prevalence of asthma and allergic symptoms. Medical records were reviewed to verify the history of infections and antibiotic prescriptions during the first 2 years of life.

## Data analysis:

Data were analyzed using appropriate statistical methods to assess the relationship between antibiotic exposure, gut microbiota composition, immune markers and health outcomes.

## Descriptive statistics:

The demographic and baseline characteristics of the study participants were summarized using means, standard deviations and percentages.

## Comparative analysis:

Differences in the gut microbiota composition between the antibiotic-exposed and non-exposed groups were assessed using independent t- tests or Mann-Whitney U tests, depending on the distribution of the data. Changes in immune markers between the two groups were analyzed using ANOVA or Kruskal-Wallis tests for multiple group comparisons.

## Longitudinal analysis:

To assess the impact of antibiotic exposure over time, mixed-effects models were used to analyze the longitudinal data, taking into account the repeated measures of microbiota composition and immune markers over the follow-up period.

Logistic regression models were used to assess the relationship between early antibiotic exposure and the development of immune- related diseases, controlling for potential confounding factors such as age, sex and socioeconomic status.

## Multivariate analysis:

Principal component analysis (PCA) or non-metric multidimensional scaling (NMDS) was applied to microbial diversity data to explore the overall differences in gut microbiota composition between the exposed and non-exposed groups.

## Ethical considerations:

The study was conducted in accordance with the principles outlined in the Declaration of Helsinki. Informed consent was obtained from all parents or legal guardians of participants. The privacy and confidentiality of participants were maintained and data were anonymized. Ethical approval was sought from the institutional review board (IRB) or ethics committee before the study commenced. Participants were informed of their right to withdraw from the study at any time without any consequence and all collected data were securely stored.

## Results:

The results of this study aimed to assess the impact of early antibiotic exposure on gut microbiota composition, immune system markers and health outcomes in children. The data were analyzed to compare the gut microbial diversity and immune function between children who had been exposed to antibiotics and those who had not been exposed. In addition, the study explored the relationship between these factors and the development of immune-related conditions over a period of 5 to 10 years. Fecal samples collected from both the antibiotic- exposed and non-exposed groups showed significant differences in gut microbial composition. The antibiotic-exposed group had a reduced diversity of gut microbiota compared to the non-exposed group. The abundance of beneficial microbes such as *Bifidobacterium* and *Lactobacillus* was significantly lower in the antibiotic-exposed group, while potentially pathogenic microbes, such as *Clostridium* and *Escherichia coli*, were more abundant. The overall microbial diversity, as measured by the Shannon Diversity Index, was significantly lower in the antibiotic-exposed group (mean 3.1) compared to the non-exposed group (mean 4.2) (Table 1). The reduction in microbial diversity was associated with an imbalance in the *firmicutes/bacteroidetes* ratio, with a higher proportion of Firmicutes in the antibiotic-exposed group (Table 2). This imbalance has been linked to increased inflammation and immune dysfunction. Immune system markers were assessed through blood samples to evaluate inflammatory cytokines, immune cell profiles and immunoglobulin levels. The antibiotic-exposed group exhibited significantly higher levels of pro-inflammatory cytokines such as TNF-α and IL-6 (Table 3). Additionally, the antibiotic-exposed group showed a higher ratio of Th17 to regulatory T cells (Tregs), indicating an imbalance in immune regulation, which is often associated with autoimmune diseases. Levels of immunoglobulins, particularly IgA, were lower in the antibiotic- exposed group, indicating potential impairment in mucosal immunity. This reduction in IgA levels was statistically significant when compared to the non-exposed group (Table 4).

The long-term health outcomes of the children were monitored for the development of conditions related to immune dysregulation, including asthma, food allergies and autoimmune diseases. The incidence of asthma and allergic rhinitis was significantly higher in the antibiotic-exposed group, with 45% of children developing asthma by the age of 5, compared to only 25% in the non-exposed group (Table 5). Additionally, the antibiotic-exposed group showed a higher prevalence of food allergies (28%) compared to the non-exposed group (12%). The prevalence of autoimmune diseases such as juvenile arthritis was also higher in the antibiotic-exposed group, further supporting the association between early antibiotic exposure and immune dysregulation. The results of this study demonstrated that early antibiotic exposure was associated with significant alterations in gut microbial composition, including reduced diversity and an imbalance in the *firmicutes/bacteroidetes* ratio. These microbial changes were associated with higher levels of pro-inflammatory cytokines and impaired immune responses, as evidenced by the reduced levels of IgA. Furthermore, the antibiotic-exposed group showed a higher prevalence of immune-related diseases such as asthma, food allergies and autoimmune disorders. These findings highlight the lasting impact of early-life antibiotic exposure on gut health and immune function, with potential implications for the development of chronic immune-related conditions later in life

## Discussion:

The findings of this study demonstrated that early childhood antibiotic exposure was associated with significant and lasting alterations in gut microbial composition, immune system markers and an increased prevalence of immune related conditions later in life. Children who received antibiotics early in life had reduced microbial diversity, shifts in key microbial phyla ratios (e.g., Firmicutes/Bacteroidetes), elevated pro inflammatory cytokines, reduced mucosal immunity markers and higher rates of conditions such as asthma, food allergies and autoimmune disorders. These results are consistent with and extend, previous research investigating the long term effects of early antibiotic exposure on the gut-immune axis. The observed reduction in gut microbial diversity aligns with findings from Lebeaux *et al.* (2022) [10] which reported that early antibiotic exposure significantly perturbs the infant gut microbiome, leading to dysbiosis that persists over time and may disrupt immune development. In their review, the authors emphasized that altered microbial communities following antibiotic use were linked with immune dysfunction and heightened disease susceptibility. Similarly, our study found that beneficial genera such as *Bifidobacterium* and *Lactobacillus* were diminished in antibiotic exposed children, supporting the assertion that early microbial depletion undermines the establishment of a robust, balanced microbiota. Our results correspond with the comprehensive review by Aires (2021) [11] which highlighted that antibiotic use during the first 1000 days of life is connected to immune dysregulation and an increased risk of allergic and inflammatory diseases. This connection between early microbial disruption and chronic immune conditions is echoed in our data, where children exposed to antibiotics exhibited higher rates of asthma and food allergies compared to non exposed peers. Broader mechanistic insights reported by Borbet *et al.* (2022) [12] indicate that early shifts in microbial populations can alter immune signaling pathways, particularly those governing the balance between pro inflammatory and regulatory responses. Our finding of elevated pro inflammatory cytokines (e.g., TNF α and IL 6) and skewed T cell ratios in antibiotic exposed children is consistent with this model of immune dysregulation originating from perturbed microbiota.

## Conclusion:

We show the long-term impact of early childhood antibiotic exposure on gut microbial balance and immune system dysregulation, with significant implications for the development of immune-related diseases. The findings highlight the need for more cautious antibiotic use during early childhood. Future research should focus on strategies to restore gut microbiota and mitigate the adverse effects of early antibiotic treatments.

## Figures and Tables

**Table 1:** Comparison of gut microbial diversity between antibiotic-exposed and non-exposed groups

**Group**	**Shannon Diversity Index (Mean ± SD)**
Antibiotic-Exposed	3.1 ± 0.5
Non-Exposed	4.2 ± 0.6
p-value	0.003

**Table 2:** Comparison of *firmicutes/bacteroidetes* ratio in antibiotic-exposed and non-exposed groups

**Group**	***Firmicutes/Bacteroidetes* Ratio (Mean ± SD)**
Antibiotic-Exposed	1.6 ± 0.3
Non-Exposed	1.2 ± 0.2
p-value	0.04

**Table 3:** Levels of pro-inflammatory cytokines in antibiotic-exposed and non-exposed groups

**Group**	**TNF-α (pg/mL, Mean ± SD)**	**IL-6 (pg/mL, Mean ± SD)**
Antibiotic-Exposed	23.5 ± 5.2	35.6 ± 7.4
Non-Exposed	14.2 ± 3.8	18.4 ± 4.2
p-value	0.002	0.001

**Table 4:** Immunoglobulin levels in antibiotic-exposed and non-exposed groups

**Group**	**IgA (g/L, Mean ± SD)**
Antibiotic-Exposed	0.55 ± 0.12
Non-Exposed	0.75 ± 0.15
p-value	0.03

**Table 5:** Prevalence of immune-related diseases in antibiotic-exposed and non-exposed groups

**Disease**	**Antibiotic-Exposed (%)**	**Non-Exposed (%)**
Asthma	45	25
Food Allergies	28	12
Autoimmune Diseases	12	6
p-value	0.02	0.05
